# Validation of primary and outcome data quality in a Swedish population-based breast cancer quality registry

**DOI:** 10.1186/s12885-024-12073-4

**Published:** 2024-03-11

**Authors:** Sofia Palmér, Carl Blomqvist, Marit Holmqvist, Henrik Lindman, Mats Lambe, Johan Ahlgren

**Affiliations:** 1 Department of Oncology, Faculty of Medicine and Health, Örebro University Hospital, Örebro University, Örebro, SE-70182 Sweden; 2 Comprehensive Cancer Center, Helsinki University Hospital and University of Helsinki, Helsinki, Finland; 3 Regional Cancer Center Central Sweden, Uppsala University Hospital, Uppsala, Sweden; 4 Department of Immunology, Genetics and Pathology, Uppsala University, Uppsala, Sweden; 5 Department of Medical Epidemiology and Biostatistics, Karolinska Institutet, Stockholm, Sweden

**Keywords:** Breast cancer, Quality registry, Validation, Data accuracy, Follow-up studies

## Abstract

**Background:**

Population-based cancer quality registries are of great importance for the improvement of cancer care. However, little is known about the quality of recurrence data in cancer quality registries. The aim of this study was to evaluate data quality in the regional Breast Cancer Quality Registry of Central Sweden, with emphasis on the validity of recorded information on recurrence.

**Methods:**

Validation by re-abstraction was performed on a random sample of 800 women with primary invasive breast cancer stage I-III diagnosed between 1993 and 2010, of which 400 had at least one registered recurrence and 400 had no registered recurrence. Registry data were compared with data from medical records. Exact agreement, correlation and kappa values, sensitivity and specificity were calculated.

**Results:**

Seven hundred forty-seven women (93%) were available for analysis. Exact agreement was high for diagnostics, tumor characteristics, surgery, and adjuvant oncological treatment (90% or more for most variables). The registry’s sensitivity was low for regional recurrence (47%), but higher for local and distant recurrence (80% and 75%), whereas specificity was overall high (≥ 95%). Combining all recurrence categories irrespective of localization improved sensitivity to 90% with a specificity of 91%. In 87% of women, the date of first recurrence according to medical records fell within ± 90 days of the date recorded in the registry.

**Conclusions:**

While the quality of data in the regional Breast Cancer Quality Registry was generally high, data accuracy on recurrences was lower. The overall precision of identifying any recurrence, irrespective of localization, was high. However, the accuracy of classification of recurrences (local, regional or distant) was lower, with evidence of underreporting for each of the recurrence categories. Given the importance of recurrence-related outcomes in the assessment of quality of care, efforts should be made to improve the reporting of recurrences.

**Supplementary Information:**

The online version contains supplementary material available at 10.1186/s12885-024-12073-4.

## Background

Cancer clinical quality registries are being increasingly used to provide data for quality assurance and research [1, 2], which makes the presence of high data quality essential [3–8]. Completeness, timeliness, comparability, and validity are considered the four key dimensions of data quality [9, 10].

Sweden, as well as other Nordic countries, has a long tradition of population-based clinical quality registries, facilitated by the unique personal identity numbers assigned to all citizens. The regional Breast Cancer Quality Registry (BCQR) of Central Sweden (the Uppsala-Örebro region) was founded in 1992 and has been part of the Swedish National BCQR since 2008. Including data on diagnostics, tumor characteristics and treatment, the quality registry represents an important complement to information in the Swedish Cancer Register, to which reporting is mandatory. Results from studies based on information in Swedish and international quality registries have had an important impact on clinical practice [11–15].

Primary tumor and treatment variables have previously been validated in different population-based regional or national cancer quality registries [16–20]. A recent validation of the Swedish National Breast Cancer Quality Registry reported high completeness (99.9%) and high comparability, while timeliness was affected by delayed reporting [21]. High validity has been reported for variables concerning diagnostics, tumor characteristics, surgery, and adjuvant treatment with an excellent agreement of approximately 95% for most treatment variables [21, 22].

Rates of recurrence and progression-free survival represent central indicators of quality in oncological care. However, little is known about the quality of recurrence data in cancer quality registries. In the three large national cancer registries in the United States, recurrence information is either not collected, or considered too unreliable to be made publicly available [23, 24]. In the Danish Breast Cancer Group (DBCG) registry, the completeness of recurrence registration was only 64–68% when patients outside of clinical trials were included [16, 18]. A validation of registry data for patients with non-invasive breast cancer in a Swedish regional BCQR showed that the proportion of registered local recurrences was 65% [25]. The value of data collected in the Swedish National and Regional Breast Cancer Quality Registries would increase substantially if it could be determined that recurrences are registered in a reliable way.

The validity of registry data on recurrence of invasive breast cancer has not previously been assessed in the national or regional Swedish BCQR. The aim of this study was to evaluate the data quality in the regional BCQR of Central Sweden, with emphasis on the validity of recorded information on local, regional and distant recurrence.

## Methods

Established in 1992, the regional BCQR of Central Sweden is a population-based registry covering a population of approximately 2 million inhabitants in seven counties. It is one of six regional registries that together constitute the national BCQR, administered by the confederation of Regional Cancer Centers (RCC). The primary aim of the registry is to include all new incident cases of breast cancer, with information on date of diagnosis, stage, biomarkers, primary treatment and recurrence. While reporting to the BCQR is not obligatory, the completeness of the primary registration of the diagnosis exceeds 99% when compared to the Swedish National Cancer Register [21], to which reporting is mandated by law. Breast cancer cases initiated by death certificates are not included in the BCQR. Follow-up registration of recurrence events was introduced in 2000, and retrospective reporting of prior recurrences was recommended when the variable was introduced. Both intended and given adjuvant treatment are registered. Before 2007, only intended treatment was reported.

For the purpose of the present data validation study, 400 women with at least one registered recurrence, and 400 women with no registered recurrence were randomly selected from the registry. For both groups, women were equally distributed over the seven counties in Central Sweden. Criteria for inclusion were women with primary invasive breast cancer diagnosed between 1993 and 2010, stage I-III disease and age ≤ 80 years. Women not treated with primary surgery and those who had emigrated were excluded.

The validity of information in the registry was assessed by re-abstraction of data from medical records, which were reviewed at seven hospitals between March, 2016 and February, 2017. Re-abstraction was performed by an oncology resident (S.P.), and a nurse with experience in registry validation. S.P. was employed by the Department of Oncology at Örebro University Hospital (a reporting unit), but had no affiliation with the BCQR or RCC. The other re-abstractor had no affiliation with either the reporting units or the BCQR. Both re-abstractors were blinded to the patients’ recurrence status as reported in the registry. Information from medical records was collected for a pre-determined set of variables and registered in a standardized manner in a specific electronic form. Re-abstracted data included diagnostics and primary surgery, tumor characteristics, adjuvant oncological treatment, and follow-up. Recurrences were categorized as local, regional, and distant. Variables included in the validation are presented in Supplementary Table S1. End of follow-up was set to December 31, 2015. The median follow-up time was 107.8 months (interquartile range: 61.2 to 161.3 months).

Date of diagnosis was defined as the date of sampling of the first diagnostic biopsy. If the exact date of diagnosis could not be determined, it was replaced with date of surgery. When information on exact date of recurrence was missing in the medical records, it was replaced with an approximate date for statistical analysis.

Any case of new invasive breast cancer in the ipsilateral breast was defined as a local recurrence. A previous diagnosis of ipsilateral invasive breast cancer warranted exclusion as the index tumor was considered a recurrence.

If the re-abstractor had difficulties classifying any variable, the case was reviewed by two experienced breast cancer oncologists (J.A., C.B.), who made the final decision.

The re-abstracted information from medical records was compared with corresponding data from the original registration. Exact agreement was calculated as well as Pearson correlation for numerical variables and Cohen’s kappa for categorical variables. Exact agreement was defined as the proportion of posts where the information in the registry was identical to re-abstracted data. For date of diagnosis, agreement was calculated both for exact date and for a time interval of ± 30 days. For date of recurrence, agreement was estimated for exact date and for time intervals of ± 30 and ± 90 days. In addition to the existing recurrence variables in the registry, a new recurrence variable (“any recurrence”) was constructed by combining all recurrence categories into one. Sensitivity and specificity were calculated for treatment and recurrence variables, including the new composite recurrence variable. For all point estimates, 95% confidence intervals (CI) were calculated. A Bland-Altman plot was constructed to assess the comparability between the information in the registry and medical records regarding time to first recurrence. Data management and random sampling was performed using SAS 9.4. Statistical analyses were performed in SPSS version 25 and R 3.5.1 and 4.3.2.

The study was approved by the Regional Ethical Review Board at Uppsala University (2015/487).

## Results

**Fig. 1:**
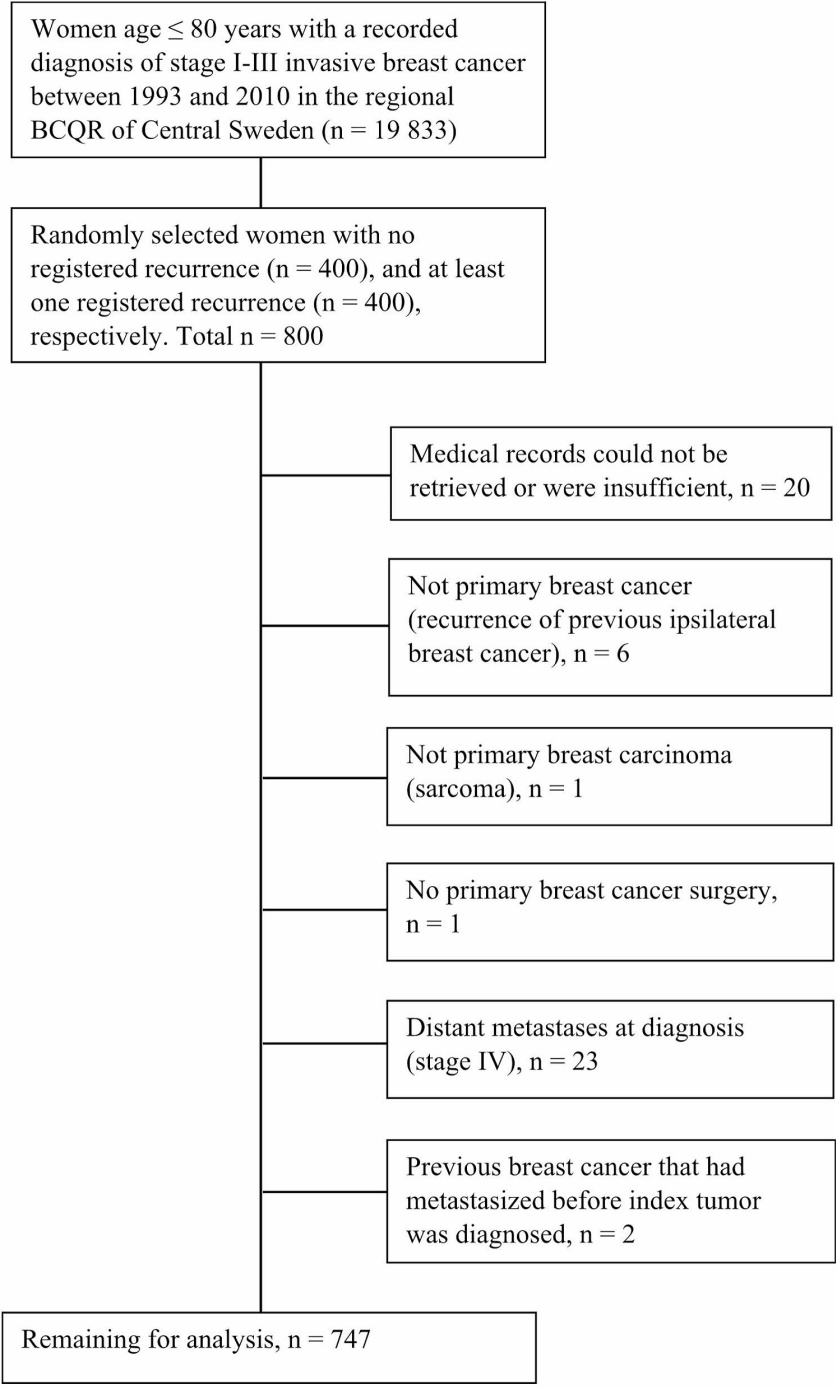
Process of selection and exclusion of patients for review

Medical records could be retrieved for 97.9% of all women, whereas 0.4% was excluded since available information in medical records were deemed insufficient for the purpose of validation. After exclusion of patients who did not fulfill inclusion criteria (3.8%) or could not be evaluated for recurrence (0.4%), 747 women remained for analysis. The process of selection and exclusion of patients for review is shown in Fig. 1. General characteristics of the study population are presented in Table 1.

**Table 1:** General characteristics of the validation sample according to the registry (*n* = 747)

Characteristics	
**Age in years at diagnosis**	
Mean (SD)	58.9 (11.6)
Median	59
Range	25–80
**Age at diagnosis**	**No of patients (%)**
< 50 years	165 (22.1)
50–59 years	217 (29.0)
60–69 years	207 (27.7)
70–80 years	158 (21.2)
**Year of diagnosis**	**No of patients (%)**
1993–1998	280 (37.5)
1999–2004	238 (31.9)
2005–2010	229 (30.7)
**Tumor size**	**No of patients (%)**
≤ 2 cm	461 (61.7)
> 2 to ≤ 5 cm	243 (32.5)
> 5 cm	38 (5.1)
Unknown	5 (0.7)
**No of metastatic lymph nodes**	**No of patients (%)**
0	406 (54.4)
1–3	171 (22.9)
4–9	92 (12.3)
≥ 10	36 (4.8)
Unknown	42 (5.6)
**Histological grade**	**No of patients (%)**
I	112 (15.0)
II	276 (36.9)
III	168 (22.5)
Unknown (missing or non-evaluable)	191 (25.6)
**Estrogen receptor (ER) status**	**No of patients (%)**
Positive	564 (75.5)
Negative	165 (22.1)
Unknown (missing or non-evaluable)	18 (2.4)
**Progesterone receptor (PR) status**	**No of patients (%)**
Positive	480 (64.3)
Negative	248 (33.2)
Unknown (missing or non-evaluable)	19 (2.5)
**HER2 status**	**No of patients (%)**
Positive	76 (10.2)
Negative	392 (52.5)
Unknown (missing or non-evaluable)	279 (37.3)
**Laterality**	**No of patients (%)**
Right	386 (51.7)
Left	345 (46.2)
Unknown	16 (2.1)
**Type of breast surgery**	**No of patients (%)**
Breast-conserving	443 (59.3)
Mastectomy	292 (39.1)
Unknown	12 (1.6)
**Sentinel node biopsy**	**No of patients (%)**
Yes	168 (22.5)
No	579 (77.5)
**Axillary dissection**	**No of patients (%)**
Yes	666 (89.2)
No	81 (10.8)
**Adjuvant chemotherapy planned**	**No of patients (%)**
Yes	257 (34.4)
No	490 (65.6)
**Endocrine therapy planned**	**No of patients (%)**
Yes	398 (53.3)
No	349 (46.7)
**Anti-HER2 therapy planned**	**No of patients (%)**
Yes	20 (2.7)
No	727 (97.3)
**Radiotherapy planned**	**No of patients (%)**
Yes	575 (77.0)
No	172 (23.0)
**Recurrence**	**No of patients (%)**
Any recurrence	369 (49.4)
Local recurrence	132 (17.7)
Regional recurrence	41 (5.5)
Distant recurrence	228 (30.5)

Completeness of data, agreement, correlation, and kappa values for variables concerning diagnostics, tumor characteristics and treatment are shown in Table 2. The numbers and proportions of concordant and discrepant cases for each variable are presented in Supplementary Tables S2-9.

**Table 2:** Completeness of data, agreement and correlation for re-abstracted variables concerning diagnostics, surgery, tumor characteristics and treatment from the BCQR

	Complete records* in registry, number (%)	Complete records* in medical records, number (%)	Complete records* in both registry and medical records, number (%)	Exact agreement between registry and medical records, number (%) (95% CI)	Correlation or Cohen’s kappa** (95% CI)
Laterality	731 (97.9%)	718 (96.1%)	703 (94.1%)	694 (98.7%) (97.6–99.3%)	0.97 (C) (0.96–0.99)
Type of breast surgery	735 (98.4%)	746 (99.9%)	734 (98.3%)	703 (95.8%) (94.1–97.0%)	0.91 (C) (0.88–0.94)
Number of investigated lymph nodes	708 (94.8%)	741 (99.2%)	706 (94.5%)	641 (90.8%) (88.4–92.8%)	0.96 (P) (0.95–0.96)
Number of metastatic lymph nodes	705 (94.4%)	707 (94.6%)	699 (93.6%)	675 (96.6%) (94.9–97.7%)	0.98 (P) (0.97–0.98)
Tumor size (mm)	742 (99.3%)	736 (98.5%)	733 (98.1%)	659 (89.9%) (87.4–91.9%)	0.90 (P) (0.89–0.92)
Chemotherapy	747 (100%)	741 (99.2%)	741 (99.2%)	688 (92.8%) (90.8–95.0%)	0.84 (C) (0.80–0.88)
Endocrine therapy	747 (100%)	740 (99.1%)	740 (99.1%)	671 (90.7%) (88.4–92.6%)	0.81 (C) (0.77–0.85)
Tamoxifen	747 (100%)	737 (98.7%)	737 (98.7%)	647 (87.8%) (85.0–90.0%)	0.76 (C) (0.71–0.80)
Aromatase inhibitor	747 (100%)	736 (98.5%)	736 (98.5%)	638 (86.7%) (84.0–89.0%)	0.50 (C) (0.41–0.58)
Anti-HER2 therapy	747 (100%)	738 (98.8%)	738 (98.8%)	727 (98.5%) (97.0–99.0%)	0.71 (C) (0.55–0.87)
Radiotherapy	747 (100%)	741 (99.2%)	741 (99.2%)	692 (93.4%) (91.0–95.0%)	0.81 (C) (0.76–0.86)

*Includes all cases with valid values for the given variable, i.e. excluding unknown and missing values

**Pearson correlation coefficient (P) for numeric variables, Cohen’s kappa (C) for categorical variables

### Diagnostics and surgery

Date of diagnosis showed an exact agreement of 16.3% (95% CI: 13.7 to 19.2%) between registry and medical records. Allowing for a time difference of ± 30 days, the agreement was 76.6% (95% CI: 73.4 to 79.6%). Agreement was high for laterality of primary tumor, type of surgery and number of investigated lymph nodes, with corresponding high kappa values and correlation coefficients. In the analysis of laterality, patients with bilateral synchronous breast cancer (*n* = 11) were excluded as the registry had no variable for bilaterality for the main part of the time period under study.

### Tumor characteristics

Agreement was high for tumor size and number of metastatic lymph nodes, with corresponding high correlation coefficients.

### Adjuvant treatment

The sensitivity, specificity, exact agreement, and Cohen’s kappa value for each treatment variable in the registry are presented in Table 3. Exact agreement was high, ranging from 90.7 to 98.5%, and Cohen’s kappa values from 0.71 to 0.84.

**Table 3:** Sensitivity, specificity, exact agreement, and Cohen’s kappa for oncological treatment variables in the registry

	Sensitivity (95% CI)	Specificity (95% CI)	Exact agreement (95% CI)	Kappa (95% CI)
Chemotherapy	88.6% (84.2–91.9%)	95.2% (92.9–96.8%)	92.8% (90.8–95.0%)	0.84 (0.80–0.88)
Endocrine therapy	88.1% (84.7–90.9%)	94.2% (91.0–97.0%)	90.7% (88.4–92.6%)	0.81 (0.77–0.85)
Anti-HER2 therapy	73.7% (49.0–91.0%)	99.2% (98.2–99.6%)	98.5% (97.4–99.2%)	0.71 (0.55–0.87)
Radiotherapy	94.7% (92.6–96.2%)	88.5% (82.0–92.6%)	93.4% (91.4–95.0%)	0.81 (0.76–0.86)

### Breast cancer recurrence

Sensitivity, specificity, exact agreement, and kappa values for each recurrence category are shown in Table 4. The concordance and discrepancy between registry and medical records for each recurrence category are presented in Supplementary Tables S10-13.

The overall agreement between registry and medical records was high for each localization of recurrence, ranging from 88.4 to 94.1%. Sensitivity was high (90.3%) when local, regional and distant recurrences were combined (any recurrence). Sensitivity was low for regional recurrence (46.9%), but considerably higher for local and distant recurrence (80.0% and 74.9%, respectively). Specificity was high for all recurrence categories, ranging between 91.2% and 97.4%. When data were split into three different time periods determined by year of first recurrence, sensitivity did not differ significantly between the time periods (Supplementary table S14).

**Table 4:** Sensitivity, specificity, exact agreement, and Cohen’s kappa for recurrence variables in the registry

	Sensitivity (95% CI)	Specificity (95% CI)	Exact agreement (95% CI)	Kappa (95% CI)
Any recurrence	90.3% (86.9–92.9%)	91.2% (87.9–93.7%)	90.8% (88.5–92.6%)	0.82 (0.77–0.86)
Local recurrence	80.0% (72.3–86.0%)	95.5% (94.0–96.8%)	92.8% (90.7–95.0%)	0.75 (0.69–0.81)
Regional recurrence	46.9% (33.0–62.0%)	97.4% (96.0–98.0%)	94.1% (92.2–95.6%)	0.48 (0.35–0.61)
Distant recurrence	74.9% (69.0–80.0%)	96.6% (94.5–97.9%)	88.4% (85.9–90.5%)	0.74 (0.69–0.79)

The agreement for date of recurrence between registry and medical records is presented in Table 5. Exact agreement on date of recurrence was low (17–28%), but increased to 87–93% when allowing a time interval of ± 90 days.

**Table 5:** Agreement in timing of recurrence events between registry and medical records

	Exact agreement (95% CI)	Agreement ± 30 days (95% CI)	Agreement ± 90 days (95% CI)
Date of first recurrence of any type	24.4% (20.0–29.4%)	67.0% (61.6–71.9%)	87.2% (83.0–90.5%)
Date of first local recurrence	27.9% (19.8–37.7%)	71.2% (61.3–79.4%)	93.3% (86.1–97.0%)
Date of first regional recurrence	17.4% (5.7–39.5%)	56.5% (34.9–76.1%)	91.3% (70.5–98.5%)
Date of first distant recurrence	25.5% (19.9–32.0%)	68.9% (62.1–74.9%)	87.3% (81.8–91.3%)

**Fig. 2:**
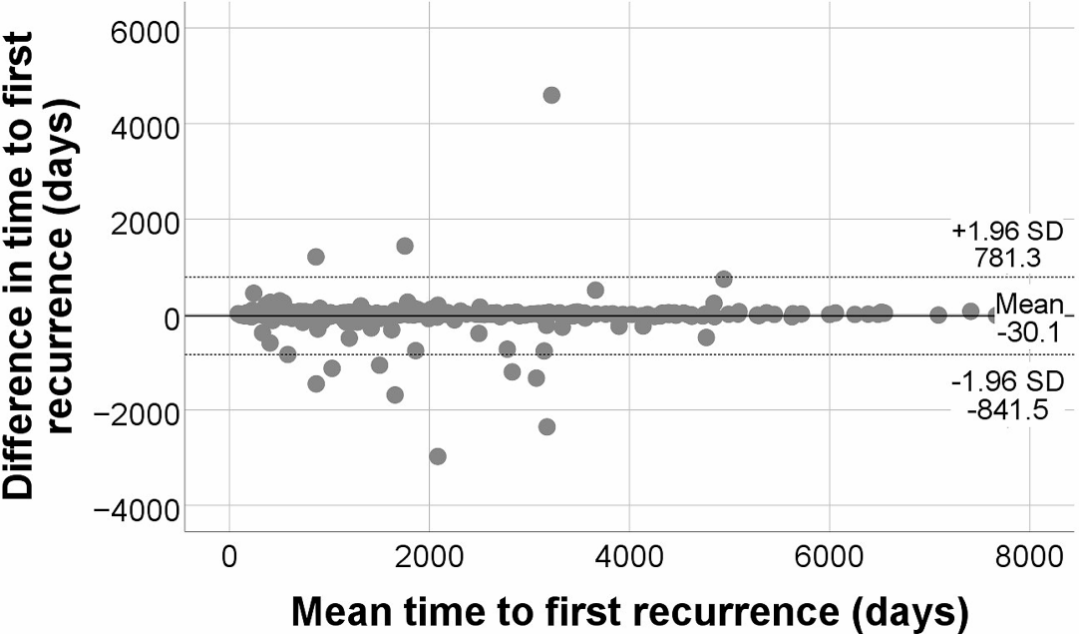
Bland-Altman plot of time to first recurrence

The comparability between time to first recurrence according to registry and medical records is illustrated in Fig. 2. The mean difference was − 30.1 days and the upper and lower 95% limits of agreement were 781.3 days and − 841.5 days, respectively. Disagreements in date of diagnosis and date of recurrence both contribute to differences in time to recurrence.

The solid line illustrates the mean, and the dashed lines show the upper and lower limits of agreement. Events with a negative difference indicate that the date of event according to the registry was later than in medical records.

## Discussion

This validation study indicates that the quality of recurrence data in the regional BCQR of Central Sweden is lower than that of tumor characteristics and treatment. Although the sensitivity of the different recurrence variables in the BCQR was low to moderate, these figures may over-estimate the precision of the registry since it is difficult to ascertain whether the first registry-reported recurrence event actually corresponds to the first recurrence documented in medical records. Combining all types of recurrence to the variable any recurrence increased sensitivity so that 90% of patients with recurrence of any type were correctly identified by the registry, and 91% of recurrence-free patients were categorized correctly. The higher precision of the composite recurrence variable indicates that there may be difficulties in the classification of recurrences.

Comparable studies with a focus on the validity of recurrence data in cancer registries are few, but have reported similar shortcomings. The Danish Breast Cancer Cooperative Group (DBCG) registry is a clinical database with high population coverage and general completeness [26], for which follow-up data has been validated in different settings [16–18, 27]. One study showed that the completeness of recurrence reporting was 93% for patients included in trials, but only 64% when all patients reported to the DBCG registry were included [16]. A validation performed in another Danish cohort [18] showed that 68% of recurrences were registered. The agreement in date of recurrence was 69% and 92% when allowing for time intervals of 1 and 4 months, respectively. In a study of women with non-invasive breast cancer based on the BCQR of Central Sweden, the proportion of reported local recurrences was 65% [25]. A recent validation of a Swedish single-institution quality registry of oral cancer showed that while 77% of recurrences were reported, 13.6% of registered recurrences were misclassified [28]. Follow-up data in the Swedish Rectal Cancer Registry seemed to have a higher completeness, with 97.6% of local recurrences and 94.4% of distant recurrences registered [29]. In a more recent assessment of recurrence registration in the Swedish Colorectal Cancer Registry, completeness differed between regions and tended to be higher after 5 years of follow-up than earlier [30].

In the present study, specificity was very high for all types of recurrence in the registry, with a slightly lower value for the composite variable any recurrence. In the Swedish Rectal Cancer Registry, the specificity was 92.3% for local recurrence and 94.8% for distant metastasis [29]. Very few false positive recurrence events were reported in the validation of recurrence data in the Swedish Colorectal Cancer Registry [30]. Neither of the other previously mentioned validation studies report any false positive recurrences [16, 18, 25, 28]. The finding of false positive recurrence events in the present study may have several possible explanations, such as potential discrepancies between the definitions of recurrence during the re-abstraction process and in the routines for reporting to the BCQR, and difficulties in determining the origin of distant metastases when more than one malignancy is present. Also, re-abstractors may have had access to more detailed information in the medical records than the clinician at the time of registration.

The results of the present study confirms that the BCQR has high data quality concerning primary tumor characteristics, treatment, and adjuvant therapy. Notably, there were some inconsistencies in the definitions of treatment variables between re-abstraction and registry. For example, pre-operative treatment was included in the definition of adjuvant treatment in the re-abstraction, while the registry variables included only post-operative treatment. Despite this discrepancy, the validity of primary tumor and treatment data remained high.

Several other population-based cancer quality registries have previously been validated for primary tumor and treatment variables with similar results. In a previous validation of the Swedish National BCQR [21], validity was high for recorded information on diagnostics, tumor characteristics, surgery, and most adjuvant treatment variables. Exact agreement was approximately 95% for radiotherapy, chemotherapy, and endocrine treatment, with kappa values between 0.82 and 0.89. Another Swedish study validated adjuvant treatment variables in the national BCQR within a cohort of younger breast cancer patients included in a trial [22] and reported comparable exact agreement and kappa values. Thus, the results of both previous validations of the BCQR are similar to the results presented in this study.

Validation studies of the Danish Breast Cancer Group registry also found high data quality on tumor characteristics and adjuvant treatment [16–18]. A validation of the Swedish National Prostate Cancer Register [19] reported high agreement for tumor characteristics and most primary treatment variables. A study validating the Swedish Quality Register of Gynecologic Cancer [20] reported a moderate to high agreement for tumor characteristics and treatment variables. An evaluation of data quality in the National Swedish Kidney Cancer Register [31] found that validity was generally high with agreements above 90% for the majority of variables and few missing values. Thus, the present study shows that the data quality for tumor characteristics and treatment in the regional BCQR is in agreement with previous validation studies in both Sweden and Denmark.

Strengths of our study include the large sample from a population-based cancer quality registry, and a thorough and systematic validation process based in re-abstraction, which is considered the most objective method of evaluating the validity of cancer registries [8, 9]. There are, however, some limitations that need to be mentioned. Although medical records are regarded as the reference, information may still be missing, invalid or open to different interpretations [8]. Another potential weakness is that the study did not include an assessment of inter-rater reliability between the two re-abstractors. In addition, the definitions of some variables differed slightly between re-abstraction and the routines for reporting to the registry. Finally, some of the registry’s variable definitions have changed during the period under study, which may have affected comparability.

Accurate information on the occurrence of recurrence including details on localization and time is important for assessment and follow-up of a country’s breast cancer care. Considering the high overall survival rate in women with breast cancer, recurrence-free survival represents an important proxy for long-term prognosis. It also reflects the burden of disease, since even a successfully treated local recurrence is associated with distress for a majority of women, and health care costs. If recurrence data are unavailable or incomplete, analysis of long-term efficacy is restricted to relative or overall survival.

## Conclusions

In conclusion, while the quality of data in the regional Breast Cancer Quality Registry of Central Sweden is generally high, data accuracy on recurrences is lower. When all recurrence categories are combined into a composite variable, the registry’s sensitivity is adequate, but there is evidence of underreporting for each of the separate recurrence categories (local, regional, and distant recurrence). Efforts should be made to improve the accuracy of recurrence data in registries considering the importance of recurrence-related outcomes in clinical practice. In addition, the findings highlight the importance of performing data validation as an integral part of the administration of all quality registries to ensure the reliability of assessment of quality of care and results from research studies.

### Electronic supplementary material

Below is the link to the electronic supplementary material.

**Supplementary Material 1: Table S1.** Variables included in the validation

**Supplementary Material 2: Tables S2-9.** Agreement between registry and medical records for variables concerning diagnostics and surgery, primary tumor and treatment

**Supplementary Material 3: Tables S10-13.** Agreement between registry and medical records for variables concerning follow-up

**Supplementary Material 4: Table S14.** Sensitivity (95% CI) of registry-reported recurrence depending on year of first recurrence

## Data Availability

The datasets generated and/or analyzed during the current study are not publicly available due to sensitive health information included in the dataset, but are available from the corresponding author on reasonable request.

## Abbreviations

BCQR: Breast Cancer Quality Registry
RCC: Regional Cancer Centers
DBCG: Danish Breast Cancer Group
ER: Estrogen receptor
PR: Progesterone receptor
HER2: Human epidermal growth factor receptor 2
