# Supplementary material for: Validation of primary and outcome data quality in a Swedish population-based breast cancer quality registry
Source: BMC Cancer. 2024 Mar 11;24:329. doi: 10.1186/s12885-024-12073-4 (PMC10926626; doi:10.1186/s12885-024-12073-4)
Supplement: Supplementary file 1 — Supplementary Material 1: Table S1. Variables included in the validation [file 12885_2024_12073_MOESM1_ESM.docx]

**Supplementary Table S1**. Variables included in the validation

| **Variable** | **Definition** |
| --- | --- |
| **Diagnostics and primary surgery** |  |
| Date of diagnosis (yyyy-mm-dd) | Earliest date of breast cancer diagnosis, verified by cytology or histology |
| Laterality (right/left/bilateral) | Laterality of primary breast tumor (bilateral if contralateral breast cancer diagnosed within 6 months from the first breast cancer) |
| Type of breast surgery  (breast-conserving/mastectomy) | Type of breast surgery; end result if more than one surgical procedure |
| Number of investigated lymph nodes | Total number of investigated lymph nodes from all axillary surgical procedures |
| **Tumor characteristics** |  |
| Tumor diameter (millimeters) | Maximal diameter of invasive tumor in millimeters |
| Number of metastatic lymph nodes | Total number of metastatic lymph nodes from all axillary surgical procedures |
| **Adjuvant or neo-adjuvant treatment** |  |
| Chemotherapy (yes/no) | Pre- or post-operative chemotherapy, at least one given treatment |
| Endocrine therapy (yes/no) | Pre- or post-operative endocrine therapy (tamoxifen, aromatase inhibitor, castration therapy), at least initiated |
| Anti-HER2 therapy (yes/no) | Post-operative trastuzumab, at least initiated |
| Radiotherapy (yes/no) | Pre- or post-operative irradiation (local and/or regional), at least initiated |
| **Follow-up** |  |
| Any recurrence (yes/no) | Breast cancer recurrence of any type |
| Date of first recurrence of any kind (yyyy-mm-dd) | Earliest date of diagnosis of first recurrence |
| Local recurrence (yes/no) | Breast cancer recurrence (invasive only) in ipsilateral breast or chest wall (unless preceded by distant metastases by ≥3 months) |
| Date of first local recurrence  (yyyy-mm-dd) | Earliest date of diagnosis of first local recurrence |
| Regional recurrence (yes/no) | Breast cancer recurrence in ipsilateral axillary, supra- or infraclavicular, or parasternal lymph nodes (unless preceded by distant metastases by ≥3 months) |
| Date of first regional recurrence (yyyy-mm-dd) | Earliest date of diagnosis of first regional recurrence |
| Distant recurrence (yes/no) | Breast cancer recurrence with any other localization |
| Date of first distant recurrence (yyyy-mm-dd) | Earliest date of first distant recurrence |
