# Supplementary material for: Validation of primary and outcome data quality in a Swedish population-based breast cancer quality registry
Source: BMC Cancer. 2024 Mar 11;24:329. doi: 10.1186/s12885-024-12073-4 (PMC10926626; doi:10.1186/s12885-024-12073-4)
Supplement: Supplementary file 2 — Supplementary Material 2: Tables S2-9. Agreement between registry and medical records for variables concerning diagnostics and surgery, primary tumor and treatment [file 12885_2024_12073_MOESM2_ESM.docx]

**Supplementary Tables S2-9**. Agreement between registry and medical records for variables concerning diagnostics and surgery, primary tumor and treatment

**Supplementary Table S2**. Laterality

|  |  | **Medical records** | | |
| --- | --- | --- | --- | --- |
|  |  | Right | Left | Missing/unknown |
| **Registry** | Right | 362 (97.1%) | 6 (1.7%) | 8 (44.4%) |
|  | Left | 3 (0.8%) | 332 (96.2%) | 9 (50.0%) |
|  | Missing/unknown | 8 (2.1%) | 7 (2.0%) | 1 (5.6%) |

**Supplementary Table S3**. Type of breast surgery

|  |  | **Medical records** | | |
| --- | --- | --- | --- | --- |
|  |  | Breast-conserving | Mastectomy | Missing/unknown |
| **Registry** | Breast-conserving | 420 (95.9%) | 23 (7.5%) | 0 (0.0%) |
|  | Mastectomy | 8 (1.8%) | 283 (91.9%) | 1 (100%) |
|  | Missing/unknown | 10 (2.3%) | 2 (0.6%) | 0 (0.0%) |

**Supplementary Table S4**. Chemotherapy

|  |  | **Medical records** | | |
| --- | --- | --- | --- | --- |
|  |  | Yes | No | Missing/unknown |
| **Registry** | Yes | 233 (88.6%) | 23 (4.8%) | 1 (16.7%) |
|  | No | 30 (11.4%) | 455 (95.2%) | 5 (83.3%) |
|  | Missing/unknown | 0 (0.0%) | 0 (0.0%) | 0 (0.0%) |

**Supplementary Table S5**. Endocrine therapy

|  |  | **Medical records** | | |
| --- | --- | --- | --- | --- |
|  |  | Yes | No | Missing/unknown |
| **Registry** | Yes | 379 (88.1%) | 18 (5.8%) | 1 (14.3%) |
|  | No | 51 (11.9%) | 292 (94.2%) | 6 (85.7%) |
|  | Missing/unknown | 0 (0.0%) | 0 (0.0%) | 0 (0.0%) |

**Supplementary Table S6**. Tamoxifen

|  |  | **Medical records** | | |
| --- | --- | --- | --- | --- |
|  |  | Yes | No | Missing/unknown |
| **Registry** | Yes | 332 (87.4%) | 42 (11.8%) | 3 (30.0%) |
|  | No | 48 (12.6%) | 315 (88.2%) | 7 (70.0%) |
|  | Missing/unknown | 0 (0.0%) | 0 (0.0%) | 0 (0.0%) |

**Supplementary Table S7**. Aromatase inhibitors

|  |  | **Medical records** | | |
| --- | --- | --- | --- | --- |
|  |  | Yes | No | Missing/unknown |
| **Registry** | Yes | 64 (43.8%) | 16 (2.7%) | 0 (0.0%) |
|  | No | 82 (56.2%) | 574 (97.3%) | 11 (100.0%) |
|  | Missing/unknown | 0 (0.0%) | 0 (0.0%) | 0 (0%) |

**Supplementary Table S8**. Anti-HER2 therapy

|  |  | **Medical records** | | |
| --- | --- | --- | --- | --- |
|  |  | Yes | No | Missing/unknown |
| **Registry** | Yes | 14 (73.7%) | 6 (0.8%) | 0 (0.0%) |
|  | No | 5 (26.3%) | 713 (99.2%) | 9 (100.0%) |
|  | Missing/unknown | 0 (0.0%) | 0 (0.0%) | 0 (0%) |

**Supplementary Table S9**. Radiotherapy

|  |  | **Medical records** | | |
| --- | --- | --- | --- | --- |
|  |  | Yes | No | Missing/unknown |
| **Registry** | Yes | 554 (94.7%) | 18 (11.5%) | 3 (50.0%) |
|  | No | 31 (5.3%) | 138 (88.5%) | 3 (50.0%) |
|  | Missing/unknown | 0 (0.0%) | 0 (0.0%) | 0 (0.0%) |
