# Supplementary material for: Validation of primary and outcome data quality in a Swedish population-based breast cancer quality registry
Source: BMC Cancer. 2024 Mar 11;24:329. doi: 10.1186/s12885-024-12073-4 (PMC10926626; doi:10.1186/s12885-024-12073-4)
Supplement: Supplementary file 3 — Supplementary Material 3: Tables S10-13. Agreement between registry and medical records for variables concerning follow-up [file 12885_2024_12073_MOESM3_ESM.docx]

**Supplementary Tables S10-13**. Agreement between registry and medical records for variables concerning follow-up

**Supplementary Table S10**. Any recurrence

|  |  | **Medical records** | |
| --- | --- | --- | --- |
|  |  | Yes | No |
| **Registry** | Yes | 336 (90.3%) | 33 (8.8%) |
|  | No | 36 (9.7%) | 342 (91.2%) |

**Supplementary Table S11**. Local recurrence

|  |  | **Medical records** | |
| --- | --- | --- | --- |
|  |  | Yes | No |
| **Registry** | Yes | 104 (80.0%) | 28 (4.5%) |
|  | No | 26 (20.0%) | 589 (95.5%) |

**Supplementary Table S12**. Regional recurrence

|  |  | **Medical records** | |
| --- | --- | --- | --- |
|  |  | Yes | No |
| **Registry** | Yes | 23 (46.9%) | 18 (2.6%) |
|  | No | 26 (53.1%) | 680 (97.4%) |

**Supplementary Table S13**. Distant recurrence

|  |  | **Medical records** | |
| --- | --- | --- | --- |
|  |  | Yes | No |
| **Registry** | Yes | 212 (74.9%) | 16 (3.4%) |
|  | No | 71 (25.1%) | 448 (96.6%) |
