# Supplementary material for: Validation of primary and outcome data quality in a Swedish population-based breast cancer quality registry
Source: BMC Cancer. 2024 Mar 11;24:329. doi: 10.1186/s12885-024-12073-4 (PMC10926626; doi:10.1186/s12885-024-12073-4)
Supplement: Supplementary file 4 — Supplementary Material 4: Table S14. Sensitivity (95% CI) of registry-reported recurrence depending on year of first recurrence [file 12885_2024_12073_MOESM4_ESM.docx]

**Supplementary Table S14.** Sensitivity (95% CI) of registry-reported recurrence depending on year of first recurrence

|  | **Any recurrence** | **Loco-regional recurrence** | **Distant recurrence** |
| --- | --- | --- | --- |
| 1993-1999 | 90.7% (82.0 – 95.4%) | 84.6% (66.5 – 93.8%) | 78.8% (67.5 – 86.9%) |
| 2000-2007 | 88.8% (82.8 – 92.9%) | 73.1% (61.5 – 82.3%) | 71.4% (62.7 – 78.8%) |
| 2008-2015 | 91.7% (86.1 – 95.2%) | 83.8% (73.3 – 90.7%) | 76.5% (67.2 – 83.8%) |
